# Animal models of listeriosis: a comparative review of the current state of the art and lessons learned

**DOI:** 10.1186/1297-9716-43-18

**Published:** 2012-03-14

**Authors:** Karin Hoelzer, Régis Pouillot, Sherri Dennis

**Affiliations:** 1 U.S. Food and Drug Administration, Center for Food Safety and Applied Nutrition, 5100 Paint Branch Parkway, College Park, MD 20707, USA

## Abstract

Listeriosis is a leading cause of hospitalization and death due to foodborne illness in the industrialized world. Animal models have played fundamental roles in elucidating the pathophysiology and immunology of listeriosis, and will almost certainly continue to be integral components of the research on listeriosis. Data derived from animal studies helped for example characterize the importance of cell-mediated immunity in controlling infection, allowed evaluation of chemotherapeutic treatments for listeriosis, and contributed to quantitative assessments of the public health risk associated with *L. monocytogenes *contaminated food commodities. Nonetheless, a number of pivotal questions remain unresolved, including dose-response relationships, which represent essential components of risk assessments. Newly emerging data about species-specific differences have recently raised concern about the validity of most traditional animal models of listeriosis. However, considerable uncertainty about the best choice of animal model remains. Here we review the available data on traditional and potential new animal models to summarize currently recognized strengths and limitations of each model. This knowledge is instrumental for devising future studies and for interpreting current data. We deliberately chose a historical, comparative and cross-disciplinary approach, striving to reveal clues that may help predict the ultimate value of each animal model in spite of incomplete data.

## Table of contents

1. Challenges in the study of listeriosis

2. Pathophysiology of infections with *Listeria monocytogenes*

3. Listeriosis in humans

3.1 Neonatal listeriosis and pregnancy-associated listeriosis

3.2 Listeriosis in adult and geriatric patients

4. Naturally occuring listeriosis among domestic and non-domestic animals

4.1 Listeriosis in ruminants

4.2 Listeriosis in monogastric mammals other than non-human primates

4.3 Listeriosis in non-human primates

4.4 Listeriosis in birds

5. Experimental infections before recognition as a foodborne disease

5.1 Experimental infections in non-pregnant animals

5.2 Experimental infections in pregnant animals

6. Mouse models of non-pregnancy-associated listeriosis

6.1 Susceptibility differences among mouse strains

6.2 Pathogenicity differences among *L. monocytogenes *strains

7. Species-specific interactions between internalines and host cells

7.1 Interactions between E-cadherin and InlA

7.2 InlB and its three receptors

8. Other animal models of non-pregnancy-associated listeriosis

8.1. Non-pregnant rat models

8.2 Non-pregnant guinea pig models

8.3 Non-pregnant rabbits as models of listeriosis

8.4 Other non-pregnant rodent models

8.5 Non-human primates as models of listeriosis

9. Animal models of pregnancy- associated listeriosis

9.1 Non-human primates as models of pregnancy-associated listeriosis

9.2 Guinea pigs as models of pregnancy-associated listeriosis

9.3 Other rodent models of pregnancy-associated listeriosis

10. Geriatric models of listeriosis

11. Conclusions and lessons learned

11.1 Consequences for modeling *L. monocytogenes *dose-response

12. Competing interests

13. Authors' contributions

14. Acknowledgements

15. Endnotes

16. References

## 1. Challenges in the study of listeriosis

Listeriosis, caused by the gram-positive, facultative intracellular bacterium *Listeria monocytogenes*, is one of the leading causes of death due to foodborne illness in the industrialized world [1,2]. Listeriosis is a relatively rare but very serious disease, with an estimated hospitalization rate that exceeds 90% and a mortality rate of approximately 15-30% [1,2]. In the United States, around 1600 human cases of invasive listeriosis occur each year, resulting in roughly 1455 hospitalizations and 255 deaths [1]. Listeriosis occurs almost exclusively in high-risk population subgroups such as pregnant women and their fetuses or infants, the elderly, or immune compromised individuals, but, as will be discussed below, clinical manifestations differ strongly among population subgroups [3-5].

Since volunteer feeding studies do not represent a viable option, the current understanding of listeriosis is mainly based on epidemiological data, clinical case reports, and the study of animal models [2,6-9]. Animal models have been of particular importance because listeriosis incidence is very low and the relatively long incubation period (i.e., average 2 to 4 weeks) complicates the reliable identification and characterization of food vehicles [2,6,9,10]. Optimal animal models closely resemble the respective infection process in humans, reliably lead to the infection endpoint of interest, allow for sufficient replicates to capture biological variability and to minimize uncertainty, and meet economic as well as ethical constraints. To date, no optimal animal model of listeriosis has been established and emerging knowledge about physiological differences among animal species has raised concerns about the direct relevance of most animal models for human disease [11]. Listeriosis has traditionally been studied in mice, but a variety of other species such as non-human primates, gerbils and guinea pigs have also occasionally been used and these species may prove preferable to mice [12-19]. Most studies have concentrated on pregnancy-associated disease or neonatal infection, while studies in non-pregnant adult animals have primarily focused on septicemia [12-19]. Geriatric models in species such as mice, rats and guinea pigs are available and some experiments have been performed in artificially immune suppressed animals, but as will be discussed below the extrapolation to human disease is challenging and the use of these models in the study of listeriosis has remained limited [20,21]. For these reasons it is largely unclear how relevant current listeriosis models are for meningitis and hosts with predisposing factors such as old age or immune defects.

Since the pathophysiology of infection is crucially important for the data discussed here, we will begin our review and discussion of animal models with a short summary of the pathophysiology of *L. monocytogenes *infection. This will be followed by a brief overview of naturally occurring clinical listeriosis in humans and different animal species and, after that, a discussion of the extensive literature on animal models of listeriosis. Since many questions about the pathogenic potential of *L. monocytogenes *in reptiles, amphibians, fish, crustaceans and other invertebrate species remain and the relevance of these models for human disease therefore appears questionable [22,23], these animals will not be explicitly discussed here even though they have occasionally been used as models of *L. monocytogenes *infection. Studies of listeriosis differ in host species and life stage, in whether they evaluate clinical symptoms or colonization of internal organs, and considerable experimental differences complicate comparison across studies even further. It is difficult to identify defensible, globally applicable and objective criteria by which to rank the scientific merit of these highly diverse studies. We therefore provide the reader with a comprehensive overview of the available scientific literature, synthesizing the medical, veterinary, immunological, microbiological and biomedical literature pertinent to the scientific value of animal models of listeriosis. Where possible, we point out important experimental details that may impact the interpretation of results, and, recognizing the immense variability across studies, we do not strive to make direct comparisons across studies. Therefore, wherever direct comparisons are made in the text, these are based on experiments that have been conducted as part of the same study and under identical experimental conditions if possible. We also point out instances where multiple independent studies found consistent or contradictory results. We therefore provide a synopsis of the currently available data and the weight of scientific evidence.

## 2. Pathophysiology of infections with *listeria monocytogenes*

Critical evaluation of the adequacy of animal models and comparisons across disease endpoints require a clear understanding of the underlying pathophysiology in humans and animals. However, numerous questions about the pathophysiology of infections with *L. monocytogenes *have remained despite longstanding concerted research efforts. The study of the pathophysiology of listeriosis has been complicated by the fact that *L. monocytogenes *can enter professional phagocytic cells such as dendritic cells through phagocytosis while direct entry into nonprofessional phagocytic cells, for example enterocytes or hepatocytes, is receptor-mediated, using caveolin-dependent or clathrin-mediated endocytosis, and dissemination within organs appears to occur mainly through direct, actin dependent spread from cell to cell [6,11].

**Figure 1:**
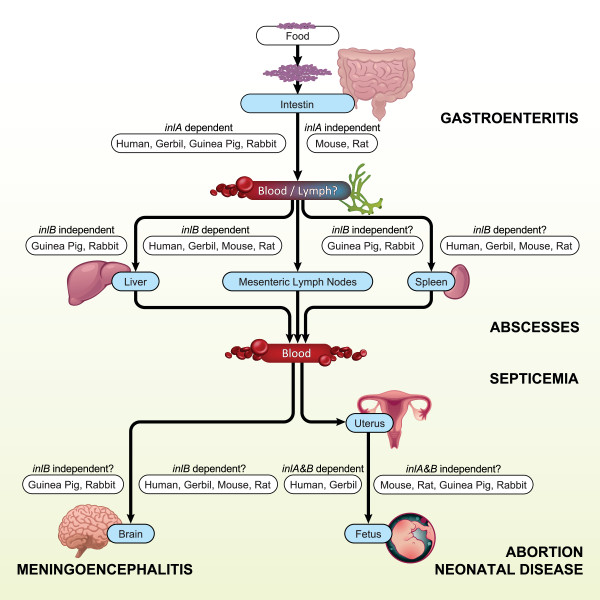
**Physiological differences among laboratory animal species as well as humans and their importance in *L. monocytogenes *infection**.

The intestine is the primary port of entry for *L. monocytogenes*, but questions about the exact mechanisms by which *L. monocytogenes *transgresses the intestinal barrier remain and clear differences among host species seem to exist (Figure 1) [6,24]. In host species deficient of functional E-cadherin such as mice (see later sections on the discovery of species-specific differences in the importance of *inlA *and *inlB *for details), *L. monocytogenes *is thought to translocate through the intestinal wall by gaining access into M-cells, phagocytic cells in the Peyer's patches of the ileum, despite some remaining controversies about the details of this process [24-27]. In species such as humans or guinea pigs that possess functional E-cadherin, *L. monocytogenes *is on the contrary, thought to primarily invade the epithelium of the intestinal villi, followed by bacterial replication in the underlying lamina propria [24,28]. *L. monocytogenes *then rapidly translocates across the intestinal barrier, without a need for bacterial replication in the intestinal wall, so that bacteria often reach the liver and spleen within minutes of oral inoculation [6]. However, extensive bacterial replication in the intestinal wall can occur during the intestinal phase of infection, and in these cases bacteria appear to move among cells of the intestinal wall via direct spread, caused by actin polymerization that is mediated by *L. monocytogenes *virulence factor *ActA *[6,28,29]. The development of lasting mucosal immunity in response to infections with *L. monocytogenes *is currently still subject to debate [30]. However, in the intestinal wall, the presence of *L. monocytogenes *stimulates dendritic cells, resident macrophages and lymphocytes, and leads to an increase in the levels of Th1-type cytokines, NF-kB, and interleukin-15 (IL-15) [6,25,31-33]. Immune responses are therefore clearly already elicited during the intestinal stage of infection.

After crossing the intestinal barrier, *L. monocytogenes *spreads to the liver, spleen and mesenteric lymph nodes, probably at least partially inside infected dendritic cells [6,24,33]. The majority of the invading bacteria become trapped in the liver and are therefore rapidly cleared from the circulatory system, followed by inactivation through immune cells such as Kupffer cells, other mononuclear phagocytic cells, neutrophils, dendritic cells and natural killer cells even though many aspects of this process have so far remained elusive [34,35]. Surviving bacteria replicate in hepatocytes, but questions about the mechanisms by which *L. monocytogenes *gains entry into these cells remain [6]. In host species with functional MetC receptors such as humans or mice (see later sections on the discovery of species-specific differences in the importance of *inlA *and *inlB *for details), *L. monocytogenes *appears to directly invade hepatocytes, probably through the Disse space after penetrating the endothelium that lines the liver sinusoids [6,36-39]. In other species, for instance guinea pigs, that do not possess functional MetC receptors, *L. monocytogenes *is thought to invade hepatocytes through cell-to-cell spread from infected Kupffer cells [6,37]. Further dissemination of *L. monocytogenes *within the liver parenchyma probably again occurs through direct, actin-mediated cell-to-cell spread [6]. Infected hepatocytes respond to infection with *L. monocytogenes *by secreting chemoattractants that recruit neutrophils to the site of infection and by initiating apoptosis, resulting in the development of typical multifocal granulomas in the liver parenchyma [6].

The remaining circulating bacteria are rapidly cleared through resident macrophages in the spleen, even though inactivation may be less efficient than in the liver and extensive bacterial replication occurs in the liver and spleen during early stages of infection [6,40]. Notably, the spleen plays a dual role in *L. monocytogenes *pathophysiology; despite initially increasing susceptibility to infection, the spleen is indispensable for the development of subsequent adaptive immune responses [41]. *L. monocytogenes *is initially ingested by macrophages and dendritic cells located in the marginal zone of the spleen, followed by translocation into the white pulp [42,43]. In the white pulp, *L. monocytogenes *induces wide-spread apoptosis, accompanied by the development of microscopic abscesses consisting of macrophages, neutrophils and apoptotic lymphocytes [44]. This process appears to be required for priming anti-Listeria cytotoxic T-cell (CTL) responses [41]. In species that possess functional MetC receptors, *L. monocytogenes *also appears to be able to enter splenocytes in an *inlB*-dependent manner, but the precise mechanisms have so far not been revealed [45].

If the infection is not controlled at this stage, for instance because of severe immune suppression, a secondary bacteremia develops, followed by dissemination of *L. monocytogenes *to a variety of secondary organs [6]. During this process *L. monocytogenes *can gain access to sanctuary sites by transgressing the blood-brain barrier or the placental barrier in pregnant hosts. Numerous questions about the exact mechanisms by which *L. monocytogenes *transgresses these barriers remain [6,46]. *L. monocytogenes *appears capable of directly invading endothelial cells including those located in the blood-brain barrier in an *inlB*-dependent manner, but in MetC deficient species, entry into endothelial cells probably occurs indirectly, mediated by direct cell-to-cell spread from phagocytic cells such as macrophages [47-51]. *L. monocytogenes *can replicate within endothelial cells and probably directly spreads to neighboring cells in an ActA dependent manner [50]. While *L. monocytogenes *is capable of directly invading neuronal cells, invasion of neurons in the central nervous system is thought to predominantly occur through direct spread from infected macrophages or microglial cells [47]. Infected macrophages may also play a direct role in transgressing the blood-brain barrier through a so-called "Trojan-horse" mechanism [52]. Many questions about how *L. monocytogenes *transgresses the placental barrier have so far also remained unanswered, but bacteria can probably cross the endothelium of the maternal blood vessels, followed by entry into the fetal circulatory system of the placental villi [6]. In animal species that possess both functional E-cadherin and MetC, this process appears to be both InlA and InlB dependent [12]. In species deficient in either of these receptors, however, crossing of the placental barrier appears neither InlA nor InlB dependent, and presumably occurs through direct cell-to-cell spread [12,53] (see section on pregnant animal models for further details on the impact of pregnancy on listeriosis). Clinical listeriosis generally develops as *L. monocytogenes *spreads to and invades secondary organs, predominantly the brain and placenta.

## 3. Listeriosis in humans

### 3.1 Neonatal listeriosis and pregnancy-associated listeriosis

Pregnancy-associated cases are thought to contribute to between 16 and 27% of invasive listeriosis cases, and often result in abortion, stillbirth or premature labor [54]. Since cases of spontaneous abortion or stillbirth are not routinely tested for listeriosis the fraction of fetal losses attributable to listeriosis is currently unknown, but the fetal mortality rate among women diagnosed with listeriosis is thought to be between 16 and 45% [55]. Listerial infection of the mother during pregnancy is often but not always associated with infection of the fetus [56,57]. One literature review, for instance, found that 20% of reported pregnancy - associated cases resulted in spontaneous abortion or stillbirth and 68% of the remaining cases (i.e., 54% of all pregnancy-associated cases) resulted in neonatal infection, indicating that fewer than 30% of pregnancy-associated cases neither led to abortion or stillbirth nor to neonatal infection [55,58]. Even though possible at any point during pregnancy, listeriosis is most frequently reported during the third trimester [2]. Clinical manifestations, apart from mild flu-like prodromal symptoms, are rarely reported in otherwise healthy women, but complications such as meningoencephalitis or endocarditis have occasionally been described, primarily in pregnant women with preexisting comorbidities [2,54,59]. Purulent villitis and microabscesses are common histopathological findings in the placentas of pregnancy-associated cases, occasionally associated with chorioamnionitis [60]. Twin pregnancies may potentially be associated with an increased risk of listeriosis, but the underlying biological determinants so far remain largely unclear [61].

Two distinct forms of listeriosis are recognized among neonates. Early onset disease, caused by infection in utero, occurs during the first week of life [2]. Neonates are often delivered pre-term, with low birth weight, and present septicemia, pneumonia, and occasionally meningitis during the first days of life [2,60,62]. Pustular skin lesions and multifocal microabscesses in the lungs, liver and spleen of affected infants are pathognomonic findings in early onset listeriosis, causing this manifestation to be commonly referred to as "granulomatosis infantiseptica" [60,61]. Early onset listeriosis has a poor prognosis, with a case-fatality rate of 20-30%, and surviving infants often develop sequelae [59].

Late onset disease, on the contrary, is typically characterized by meningitis, sometimes accompanied by other symptoms such as fever, colitis and diarrhea [62]. This manifestation generally occurs in infants 7 to 20 days after birth [61]. In these cases, pregnancy was usually uneventful, carried to term, and infants appeared healthy at birth [59]. The source of infection often remains unclear, but perinatal infections through contact with the birth canal, maternal feces, or the home environment have been suggested, as well as nosocomial transmissions [59,62-65]. Case-fatality rates for late onset listeriosis have been estimated at approximately 10%, and neurological sequelae have occasionally been described in surviving infants [61].

### 3.2 Listeriosis in adult and geriatric patients

Meningitis or meningoencephalitis and septicemia are the most common clinical manifestations of invasive listeriosis in adults and are generally associated with comorbidities such as malignancies, immunosuppressive therapies, alcoholism, hepatopathies, renal failure, HIV infection, diabetes mellitus, autoimmune disorders or hemochromatosis [61,66-68]. Septicemia occurs in an estimated 21-43% of cases, and is often manifested as fever, nausea, vomiting and myalgia [66]. These conditions can be complicated by disseminated intravascular coagulation, respiratory distress and multi-organ failure [66].

Headache, nausea, high fever, stiff neck, confusion, lethargy and less frequently ataxia, tremor and seizures are typical clinical symptoms associated with listerial meningitis or meningoencephalitis [66,68,69]. Typical histopathological findings include suppurative meningitis with purulent exudate concentrated around the brain stem, and white-gray foci of microabscesses in the meninges and occasionally brain cortex [69]. In approximately 10% of cases, *L. monocytogenes *affects the cortex parenchyma, resulting in encephalitis and abscess formation which is typically manifested as cognitive dysfunction and altered consciousness [68]. Histological findings in the brains of patients with listerial encephalitis include perivascular microabscesses, multifocal vasculitis, and perivascular cuffing [69]. Case - fatality rates of 15-27% have been reported for listerial meningitis or meningoencephalitis and case-fatality rates of up to 59% have been mentioned for *L. monocytogenes *brain abscesses [67,68].

In healthy adults, listeriosis is typically manifested as gastroenteritis, a mild, self-limiting condition characterized by fever, diarrhea, abdominal cramps, nausea and vomiting, headache, myalgia and arthralgia [2,70]. However, rhombenchephalitis, a rare but very severe form of listerial encephalitis, also occurs predominantly in adults without classical comorbidities [61,71]. Rhombenchepahlitis is characterized by a typical biphasic course - a prodromal stage with flu-like symptoms such as fever, headache, myalgia, nausea and vomiting, followed by the sudden onset of unilateral or bilateral paralysis of cranial nerves, ataxia, vertigo and impaired consciousness [61,71]. Death often occurs due to respiratory or cardiac failure, and sequelae are common in survivors [71]. Perivascular cuffing and microabscesses in cerebellum and medulla oblongata are common associated histological findings [61,71].

A variety of atypical manifestations of listeriosis, involving for instance the eye, joints, bones, heart or skin have also been documented in rare cases, and cutaneous infections represent occupational hazards for veterinarians during obstetric manipulations [55,72].

## 4. Naturally occuring listeriosis among domestic and non-domestic animals

*L. monocytogenes *was first described by Murray et al. in 1926 who isolated the bacterium from the livers of clinically sick rabbits and guinea pigs [23,73]. Since then listeriosis has been recognized as a disease of mammals and birds, and as a potential zoonosis [6,74-77]. During the 1980s several large outbreaks among humans led to the recognition of *L. monocytogenes *as an important foodborne pathogen, shifting the focus from a veterinary to a human public health problem [6,78].

### 4.1 Listeriosis in ruminants

Even though *L. monocytogenes *can infect a wide variety of animal species, listeriosis is primarily a clinical disease of ruminants, which can also be caused by *L. ivanovii*, a *Listeria *species non-pathogenic for humans and other animal species [23,55]. Sheep appear to be particularly susceptible to infection, but listeriosis is also common in a variety of other polygastric species and *L. monocytogenes *has for instance been isolated from cattle, goats, llamas, alpacas, deer, reindeer, antelopes, water buffalos and moose [23,55,79-81]. It is worth mentioning that bacterial shedding in the absence of clinical symptoms has occasionally been observed [55,82,83].

Listeriosis represents one of the most common etiologies for encephalitis among adult ruminants [55]. Ruminants affected by encephalitis generally show marked neurological symptoms including ataxia, "circling", opisthotonus, and paralysis of cranial nerves, combined with hyperthermia, anorexia and depression [84]. Encephalitis is the most common clinical manifestation of listeriosis in ruminants, while large epidemics of third trimester abortions, typically manifested as stillbirth, as well as atypical manifestations such as conjunctivitis have also repeatedly been described [55,84,85]. With the exception of neonates and young ruminants, septicemia is unusual, but can result in mastitis, gastro-enteritis, hepatitis, or pneumonitis [55,86]. Notably, in a given affected herd listeriosis generally exhibits a single clinical manifestation [55,86].

Listeriosis occurs seasonally among ruminants, with the highest incidence in winter and early spring, and appears strongly associated with ingestion of spoiled silage [55,87]. It has been suggested that *L. monocytogenes *may cause rhombencephalitis in ruminants through centripetal migration along cranial nerves, particularly the trigeminal nerve, followed by multiplication in pons and medulla oblongata [6,55]. Consistent with this hypothesis, changes in dentation and other lesions in the oral cavity as well as on the lips, nostrils or conjunctiva appear to be predisposing factors for listeriosis in ruminants [55]. Typically, histopathological findings in ruminants with rhombencephalitis are unilateral, located in the brain stem, particularly pons and medulla oblongata, and include perivascular cuffing and multifocal microabscesses, generally without involvement of meninges or choroid plexus [55,84,88]. These lesions clearly resemble those observed in humans affected by rhombencephalitis [88]. Septicemic cases among ruminants are characterized by multifocal necrosis of the liver, spleen, and potentially other organs [55,84]. Placentitis and endometritis are typical findings associated with abortions [84].

### 4.2 Listeriosis in monogastric mammals other than non-human primates

Clinical listeriosis is relatively rare in most monogastric mammals such as dogs, cats, horses and pigs, but appears more common in rodents and lagomorpha, where listeriosis was first described [23,73,79]. Notably, *L. monocytogenes *has also been isolated from clinically healthy monogastric mammals [55,82]. Listeriosis in monogastric mammals is typically manifested as septicemia [55,89]. Abortion, meningoencephalitis and other manifestations such as conjunctivitis are also possible, but their relative frequency differs by animal species [55,90]. Large outbreaks of listeriosis have been reported among colonies of captive rodents and lagomorpha, including chinchillas, rabbits, rats and guinea pigs [55]. Contaminated feed such as hay or sugar beets was implicated as the outbreak vehicle in many of these outbreaks, and coprophagy may have contributed to some of the outbreaks [55]. *L. monocytogenes *has also been isolated from a variety of other rodents and lagomorpha including gerbils, bush-tailed jirds, mountain hares, European hares, Japanese hares, voles, field mice, muskrats, shrews, capybaras, and squirrels, as well as rock hyrax and other mammals in zoological exhibits, but the association with clinical disease is not in all cases clear [23,91-100]. In some of the outbreaks reported among rodents and lagomorpha disease progression was peracute, and death occurred prior to the development of pronounced pathological lesions [55,101]. In other cases septicemia and neurological symptoms such as torticollis and ataxia dominated, even though metritis and abortion have also been described [102,103]. For currently unknown reasons chinchillas and rabbits appear particularly susceptible to infection [73,101,103-105]. Abortion and metritis are quite common, especially in chinchillas, and are often associated with gastro-intestinal symptoms such as diarrhea, constipation, intestinal invaginations or prolapsed rectum [104]. Common histophathological lesions include multifocal necrosis of the liver and necrotizing endometritis [94,102].

### 4.3 Listeriosis in non-human primates

A small number of listeriosis cases among captive non-human primates have been described [106-108]. In addition, *L. monocytogenes *has been isolated from feces of wild monkeys in Japan and *Listeria *from the blood of wild baboons in Africa, but the absence or presence of clinical symptoms in the animals was not reported and in the latter case the *Listeria *species was not identified [96,109]. Clinical manifestations of listeriosis as septicemia, meningoencephalitis and abortion have been observed in captive non-human primates, with reported neurological symptoms including stiffness of the neck and paralysis of the facial nerves [106,108,110,111]. Post-mortem examination of a non-human primate affected by purulent meningoencephalitis revealed perivascular cuffing and mononuclear cell infiltration, while focal hepatic necrosis and placentitis were reported in a case of perinatal septicemia, and necrosis of the placental villi as well as multifocal necrosis of several fetal organs was described in a case of abortion [107,108,110].

### 4.4 Listeriosis in birds

Clinical listeriosis in birds is rare, and seems to frequently represent a secondary infection which has been associated with a variety of viral, bacterial or parasitic diseases as well as tumors [23,55]. Young birds are more susceptible to disease than adult birds, and susceptibility differs among avian species [23,76]. *L. monocytogenes *has been isolated from a wide variety of domestic and wild birds including chickens, geese, ducks, turkeys, pigeons, canaries, parrots, eagles, owls and partridges [76]. Similar to observations in mammals, bacterial shedding in the absence of clinical symptoms has occasionally been described [23]. Disease in birds is most commonly manifested as septicemia, resulting in focal necrosis of the liver, spleen, heart, kidneys, lungs, air sacks, intestine, oviduct or cornea [23,76]. Listerial meningoencephalitis is uncommon among birds [23,55]. Affected fowl exhibit typical central nervous system symptoms including torticollis, tremor, and paralysis of the legs or wings [23,55]. Post-mortem examination of affected birds often reveals perivascular cuffing and focal necrosis in the cerebellum and medulla oblongata, which is frequently accompanied by septicemic lesions in the liver and spleen [55].

## 5. Experimental infections before recognition as foodborne disease

### 5.1 Experimental infections in non-pregnant animals

The first report of experimental inoculations with *L. monocytogenes*, in rabbits, dates back to the first study describing this pathogen in 1926 [73]. In the following decades, before *L. monocytogenes *was recognized as a major foodborne pathogen, numerous animal experiments were performed in a large variety of species including mice, rats, rabbits, guinea pigs, dogs, cats, pigs, ruminants and non-human primates [23]. Because pathogenesis and in prticular infection routes were essentially unknown, studies often compared a large number of exposure routes (e.g., oral, gastric, intraveneous, intraperitoneal, intracerebral, subcutaneous, submucosal, conjunctival, vaginal and nasal), and different studies occasionally reported seemingly contradictory results [23,112,113]. Notably, it is extremely difficult to experimentally produce listeriosis in non-pregnant animals that resembles naturally occurring disease proves extremely difficult [23]. Encephalitis or meningoencephalitis are extremely difficult to evoke unless bacteria are instilled directly in the cerebrum, partially because animals tend to die before meningitis can develop [23]. Intravenous, intraperitoneal and intracerebral routes of exposure reliably lead to disease in non-pregnant animals, but their relevance for naturally occurring disease appears questionable [23]. Respiratory routes of infection using aerosolized inoculum are generally efficient means of inoculating non-pregnant mice, guinea pigs, hamsters, rabbits, piglets and non-human primates, and in many experiments animals succumbed to septicemia [23,75,114,115]. In experimental infections, non-human primates developed pyrexia but recovered from aerosol exposure and gross septicemic lesions were absent upon post-mortem examination of sacrificed animals, even though some animals mounted a humoral immune response after inoculation and bacteria could be isolated from the blood of some animals post inoculation [23,115]. Except for chinchillas and certain strains of mice, oral exposure, even at high dose, rarely leads to disease in non-pregnant animals, with the exception of very young animals [23]. However, successful oral inoculation after starvation has occasionally been reported [116]. Remarkably but consistent with observations from naturally infected animals, in some instances bacteria can be isolated from experimentally inoculated animals in the absence of clinical symptoms or pathological lesions, albeit bacterial concentrations are likely low since cultures had to be kept at refrigerated temperatures for several weeks to culture *L. monocytogenes *from the animal tissues, thereby hampering the growth of background microflora and allowing the psychotropic bacteria to reach numbers sufficient for detection [23,117]. Ocular inoculation produces conjunctivitis and other eye infections in non-pregnant animals of various species including rabbits, guinea pigs and non-human primates, and exposed animals occasionally develop septicemia, meningitis or meningoencephalitis [23,118-120]. Non-human primates mostly develop mild and transient ocular symptoms while guinea pigs and rabbits develop severe symptoms and occasionally succumb to systemic infection [120]. Irrespective of exposure route or animal species, inoculation of non-pregnant animals often leads to generalized septicemia, even in ruminants, and sustained septicemia occasionally - though not reproducibly - results in meningitis or meningoencephalitis [23,113].

Importantly, the clinical manifestation of experimental infection appears highly dose-dependent; high inoculation doses tend to lead to peracute death without visible involvement of the central nervous system [23,113,115]. Focal necrosis of the liver with infiltration of mononuclear cells is a typical histopathological finding in septicemic animals, sometimes also affecting the spleen, lungs, and other organs such as the tonsils, intestinal tract or adrenal glands [23,113,121]. In general, susceptibility to infection differs with *L. monocytogenes *strain, inoculation dose, age group - with suckling mice particularly susceptible to infection-, animal species and also immune status [23,75,115,122]. For example, it was found that injecting mice with Bacillus Calmette-Guérin (BCG, a vaccine against tuberculosis) prior to *L. monocytogenes *challenge increases resistance to listeriosis, while experimentally induced stress reduces resistance, at least in hamsters, guinea pigs and possibly in lemmings [75,123]. Guinea pigs, hamsters, dogs, cats and pigs overall appear considerably more resistant to infection than rabbits and mice; in guinea pigs focal necrotic lesions appear atypical in that they are often limited to the myocardium [23,115,123].

### 5.2 Experimental infections in pregnant animals

Pregnancy-associated listeriosis has been studied in ruminants and several monogastric species using a variety of exposure routes [23]. Regardless of placentation type, gestational stage and exposure route, experimental inoculation of pregnant animals often results in abortion [23,122,124]. Oral inoculation efficiently produces abortion, as demonstrated for example in pregnant rabbits and goats [23]. Perinatal infection through vaginal contamination was also shown to be possible, but rarely occurs even under experimental conditions [23,113]. In guinea pigs and rabbits abortion following conjunctival challenge has also been reported [23,122,124].

Clinical manifestations clearly differ by gestational stage at the time of inoculation and infectious dose [122,124]. Placentitis, endometritis and focal necrosis are common findings in aborted animals, and live borne animals often succumb to septicemia or meningoencephalitis, depending on the length of the time interval between birth and the onset of disease symptoms [23,122]. Placentitis and the resulting nutritional limitations for the fetus seem to play a major role in the development of abortion. In the placentas of experimentally infected rats necrotic lesions are predominantly focused in the junctional zone of the placental disc, but often extend to the labyrinth and metrial glands, and maternal sinuses are infiltrated with monocytes and polymorphic cells [23,125]. Importantly, crossing of *L. monocytogenes *through the placental barrier in the absence of placental lesions has also been described [23,126].

## 6. Mouse models of non-pregnancy-associated listeriosis

Since the identification of *L. monocytogenes *as a major human food-borne pathogen in the early 1980s, considerable attention has been devoted to oral or intragastric routes of exposure. Infection through the oral route is thought to be the most relevant for humans but poses considerable practical challenges. For a long time, mice and to a lesser extent rats were the most popular species used to establish oral models of listeriosis, predominantly evaluating septicemic death [11,27,127,128]. The development of invasive disease in these animals is dose-dependent, but in adult animals relatively high doses are often required to invoke disease and death [11,127,128]. Despite these limitations, murine and rat models have proved instrumental in elucidating key aspects of *L. monocytogenes *infection and immunity, and for instance have allowed establishment of a correlation between reduced gastric acid levels and increased susceptibility to infection, which had been suggested through epidemiological studies in humans [128-130]. Due to the difficulty of reproducibly invoking and monitoring disease in mice after experimental inoculation, death was often chosen as the study endpoint and the dose at which 50% of inoculated mice died (i.e., median lethal dose, or LD_50_) was commonly used to compare results across studies. Other study outcomes such as bacterial concentrations in different organs or ratios of different *L. monocytogenes *strains used in the inoculum cocktail (i.e., competitive indexing), which represent commonly chosen study outcomes in most other animal species, have occasionally been used in mouse studies of listeriosis. As reported above, neurological symptoms have proven particularly difficult to evoke experimentally. However, repeated oral challenge of mice with sublethal doses (i.e., 5 × 10^9 ^cfu) has been shown to lead to the establishment of CNS symptoms, at least in some of the animals [131]. Notably, repeated dosing over a longer time period (i.e., 10 vs. 7 consecutive days) appears to result in a somewhat higher prevalence of CNS symptoms while dosing for less than 5 days does not result in the development of clinical CNS symptoms, emphasizing the potentially paramount importance of multiple dosing [131].

### 6.1. Susceptibility differences among mouse strains

The susceptibility of mice to *L. monocytogenes *infection is affected by the physiological state of the animal and differs considerably among mouse strains [132-140]. LD_50 _values for different mouse strains and exposure routes often differ by several orders of magnitude (Table 1), even though differences in experimental design (e.g., inoculums size, preparation and quantification of inoculum, animal sex and age group, number of days of follow-up after inoculation, *L. monocytogenes *strain used for inoculation, use of bicarbonate treatment, starvation or immune suppression prior to inoculation, method of LD_50 _calculation, etc.) complicate comparison across studies. Mice of strains A/J or BALB/c, for example, are considerably more susceptible to intragastric as well as intraveneous and intraperitoneal infection than mice of strain C57BL/6, with reported intragastric LD_50 _values equaling 10^6 ^and 10^8 ^cfu for mice of strains A/J and C57BL/6, respectively [137,138,141].

**Table 1:** Susceptibility of some mouse strains used as models of non-pregnancy-associated *L. monocytogenes *infection, measured as median lethal dose (LD_50_)

mouse strain	**LD**_**50 **_**range (in CFU) reported in the literature**						Recognized mouse phenotype
	oral/intragastric		intravenous		intraperitonial		
	**LD**_**50 **_**(strain)**	Reference	**LD**_**50 **_**(strain)**	Reference	**LD**_**50 **_**(strain)**	Reference	
A/J	**10**^**6 **^(a)	[137]	**10**^**3 **^(b, d)	[138,141]	**< 10**^**7 **^**- 10**^**11 **^(b, d,e, i)	[142,143]	susceptible
A/Tru × C57Bl/6^*7*^			**10**^**4 **^(b)	[144]			-
A/Tru × C57Bl/6^4^			**10**^**6 **^(b)	[144]			-
A/Tru × C57Bl/6^8^			**10**^**5 **^(b)	[144]			-
A/WySn			**10**^**4 **^(d)	[138]			-
BALB/c			**10**^**3 **^(b, d)	[138,145]	**10**^**5 **^(i)	[147]	susceptible
			**10**^**4 **^(c)	[146]			
			**10**^**5 **^(f, i)	[139]			
B10.A			**10**^**5 **^(b,d)	[138,141]			resistant
B10.D2/Sn			**10**^**5 **^(b, d)	[138,141]			resistant
CBA			**10**^**3 **^(d)	[138]			susceptible
C3HeB/FeJ					**10**^**4 **^(i)	[148]	susceptible
C57BL/6	**10**^**3 **^(b)	[145]	**10**^**3 **^(b)	[145]			resistant
	**10**^**8 **^(a)	[137]	**10**^**5 **^(b)	[149]			
	**> 10**^**10 **^(b)	[149]	**10**^**6 **^(d, f, i)	[138,139]			
C57BL/6 × BALB/c			**10**^**3 **^(b)	[42]			intermediate
			**10**^**4 **^(d)	[138]			
C57BL/6 × DBA/2 N			**10**^**4 **^(b)	[150]	**10**^**7 **^**- 10**^**9 **^(b, e, i)	[143]	intermediate
C57BL/10Sn			**10**^**5 **^(b)	[141]			resistant
DBA/2 J			**10**^**3 **^(b)	[141]			susceptible
ddY^3^	**10**^**4 **^(g)	[127]					-
ddY^4^	**>10**^**9 **^(g)	[127]					-
ICR			**10**^**5 **^(d)	[151]	**10**^**4 **^**- 10**^**7 **^(a,d,e,i)	[152]	intermediate
					**10**^**4 **^**- >10**^**8 **^(b,d,i)	[19,153]	
					**10**^**5 **^**- >10**^**9 **^(a,d,e,i)	[154]	
Inbred white (Washington State University)					**10**^**5 **^**- 10**^**7 **^(c)	[155]	-
NCR^1^	**10**^**1 **^**- 10**^**5 **^(d, e, i)	[17]			**10**^**1 **^**- 10**^**5 **^(d, e)	[17]	-
NCR	**10**^**3 **^**- 10**^**6 **^(d, e,i)	[17]			**10**^**2 **^**- 10**^**6 **^(d, e,i)	[17]	-
NMRI^5^			**10**^**4 **^(f)	[156,157]	**10**^**4 **^**- 10**^**12**^	[158]	-
			**10**^**4 **^**- 10**^**12 **^(f,g,h,i)	[158]	(f,g,h,i)		
Swiss			**10**^**5 **^(b)	[159]			-
Swiss-Webster					**10**^**1 **^**- 10**^**5 **^(i)	[115]	-
					**10**^**5 **^**- 10**^**7 **^(c)	[155]	
Swiss white					**10**^**7 **^(a,d,e)	[18]	-
Porton	>**10**^**10 **^(b,d,e,i)	[160]					-
129 Sv × C57BL/6					**10**^**4 **^(b)	[161]	intermediate
iFABP-hEcad^2^	**~10**^**10 **^(b)	[149]					transgenic
E16PmEcad^6^	n/a						transgenic

Animals represent non-pregnant adults unless stated otherwise.

^1^body weights ranging from 10 - 20 grams; ^2^(C57BL/6 J × SJL/J) background; ^3^suckling mice; ^*4*^adult mice; ^5^derived from swiss-type mouse; ^6^only competitive indexing experiments performed; ^*7*^juvenile mice; ^*8*^geriatric mice

*L. monocytogenes *strains:

(a) *L.monocytogenes *Scott A (serotype 4b); (b) *L.monocytogenes *EGD (serotype 1/2a); (c) *L. monocytogenes *10403S (serotype 1/2a); (d) human clinical isolate; (e) food isolate; (f) *L. monocytogenes *4b isolate; (g) *L. monocytogenes *1b isolate; (h) *L. monocytogenes *3a isolate; (i) other or not specified *L. monocytogenes *isolate.

The physiological or immunological determinants of these susceptibility differences have so far only been partially elucidated. After bicarbonate treatment, mice of strains BALB/c and C57BL/6 developed more marked gastric and intestinal lesions in response to oral inoculation with 10^9 ^cfu *L. monocytogenes *than mice of strains ICR, C3H and FVB, potentially indicating mouse strain-specific differences during the intestinal phase of infection [162]. Certain mouse strains probably also differ in the ability to control infection in the liver since differences in the size and frequency of hepatic lesions between mice of susceptible strain BALB/c and resistant strain C57BL/10 have been described [163].

Susceptibility differences are at least partially genetically determined: mice of strain C57BL/6 and related sublines NZB and SJL appear considerably more resistant to intravenous inoculation than mice of strains A/J, BALB/c or CBA, and LD_50 _values equaling 9 × 10^5 ^and 4 - 8 × 10^3^, respectively, have been reported [138]. Back-crossed (C57BL/6 × BALB/c) mice, on the contrary, showed intermediate susceptibility with LD_50 _values in the range of 3.4 × 10^4 ^cfu [138]. Notably, the degree of susceptibility to infection also varies among individual back-crossed animals, possibly indicating that susceptibility differences are controlled by multiple genetic loci [138,164]. Many laboratory mice strains are intentionally bred for their distinctive immunological characteristics, and these immunological differences probably represent one of the key determinants of susceptibility differences to *L. monocytogenes *infection. Susceptibility of the A/J strain and certain other strains such as DBA/2, for instance, appears to be linked to allelic variation in the *Hc *locus, which controls complement C5 levels in the mouse [133,164,165]. Yet, other *L. monocytogenes *susceptible mouse strains such as BALB/c are C5-sufficient, strongly suggesting the presence of additional susceptibility determinants [133].

Differential cytokine expression during infection likely contributes to susceptibility differences among inbred mice strains. After intravenous inoculation with 6 × 10^3 ^cfu of *L. monocytogens*, interleukin transcription levels, in particular IL-12 and IL-15, were higher in dendritic cells from spleens of C57BL/6 mice than in dendritic cells from spleens of BALB/c mice [166]. Mice of strain C57BL/6 also expressed higher INF-*γ *and GM-CSF levels in the spleen shortly after infection than mice of strain A/J, even though levels in the liver appeared similar [167]. Differences in susceptibility between C57BL/6 substrains C57BL/6 J and C57BL/6By after intravenous *L. monocytogenes *inoculation have been linked to differential *Ifnb1 *expression, with increased IFN*β *levels increasing susceptibility to infection, thus emphasizing the potentially important role of cytokine expression in strain susceptibility [132].

Sex may directly impact susceptibility of adult mice to *L. monocytogenes *infection, potentially due to differences in IL-10 expression, even though contradictory results have been reported [168]. Pasche et al., for instance, showed that, based on survival time differences after challenge, female mice of strains BALB/c, C57BL/6, C3H/HeN and CBA/J were significantly more susceptible to intravenous inoculation than concurrently inoculated male mice of the same strains, with associated p-values ranging from 0.002 to 0.05 for the different strains [168]. Mainou-Folwer, on the contrary, did not detect significant differences in LD_50 _values between male and female mice of BALB/c or C57BL/6 strains after intraveneous inoculation [139], and Cheers and McKenzie did not detect marked differences in survival between male and female mice of various strains including BALB/c, CBA and C57BL/6 [138].

Age significantly impacts susceptibility to infection. For instance, LD_50 _values for specific pathogen free (SPF) sucking mice of strain ddY after intragastric inoculation have been shown to be approximately 10^5 ^cfu lower than those for 5 week old SPF animals of the same strain [127]. Pine et al. demonstrated approximately 10 fold differences in susceptibility of 21 compared to 33 day old female mice of strain NCR after intragastric *L. monocytogenes *inoculation, regardless of the *L. monocytogenes *strain used [17]. Backcrossed (A/Tru × C57BL/6) mice 1, 8 and 24 months of age exhibited LD_50 _values of 1.6 × 10^4^, 4.0 × 10^6 ^and 1.6 × 10^5 ^cfu, respectively, when inoculated intravenously with *L. monocytogenes *strain EGD, even though age differences were not observed when mice were inoculated with low bacterial doses [144]. A variety of immunological, genetic and physiological determinants therefore impact susceptibility of mice to *L. monocytogenes *infection, and may complicate comparisons across studies.

### 6.2. Pathogenicity differences among *L. monocytogenes *strains

Regardless of the mouse strain or the host's physiological status, susceptibility of mice to intragastric as well as other routes of inoculation clearly depends on the *L. monocytogenes *strain used for inoculation (Table 1). For example, when mice of the BALB/c strain were inoculated through the intragastric route with 2 × 10^9 ^cfu of different *L. monocytogenes *strains, bacterial loads in internal organs clearly differed among *L. monocytogenes *strains [169].

*L. monocytogenes *strains are known to differ in their pathogenicity for humans, and the molecular determinants of these pathogenicity differences are beginning to be elucidated [55]. Notably, while a large fraction of strains isolated from food sources contain a premature stop codon in the *inlA *gene that attenuates their virulence, such attenuated strains are rarely isolated from human cases, and outbreak strains generally express full-length InlA [55]. Similar to these pathogenicity differences among humans, mice appear to be more susceptible to infection with human outbreak strains than with isolates from food sources. For example, systemic infection was significantly (*p *< 0.01) more likely in mice of strain A/J inoculated with 10^6 ^CFU of epidemic *L. monocytogenes *strains from human outbreaks than in mice inoculated with similar amounts of *L. monocytogenes *strains from environmental or food sources [136]. Similarly, after challenging adult mice of the NCR strain with different *L. monocytogenes *isolates from clinical and food source, LD_50 _values ranged from 10^3 ^to 10^5 ^cfu [17]. In outbred mice of the ICR strain, intragastric inoculation with 10^6 ^cfu of *L. monocytogenes *Scott A, a serotype 4b strain, led to pathologically more severe lesions than inoculation with equal amounts of *L. monocytogenes *strain EGD, a serotype 1/2a strain, again reflecting pathogenicity trends among humans [170]. Surprisingly, pretreating mice with sodium bicarbonate to neutralize the stomach pH appeared to have a considerably more pronounced effect on infections with strain EGD than on infections with the Scott A strain [170]. Similarly pronounced differences among *L. monocytogenes *subtypes have been observed in pregnant gnotobiotic BALB/c mice after oral challenge, with a serotype 3 strain apparently unable to colonize the murine gut and invade the host [154]. Back-crossed (C57BL/6 × DBA/2) mice, inoculated intragastrically with 2 × 10^9 ^cfu of hemolytic or non-hemolytic *L. monocytogenes *strains showed considerably different bacterial loads in mesenteric lymph nodes, spleen and liver, again emphasizing the similarities in *L. monocytogenes *strain specific pathogenicity between mice and humans [171]. Properties of the inoculum strain, in addition to immunological and physiological properties of the host, therefore seem to significantly impact the outcome of experimental *L. monocytogenes *inoculations in mice, and *L. monocytogenes *strain specific pathogenicity differences in mice may at least partially reflect pathogenicity differences in humans.

## 7. Species-specific interactions between internalines and host cells

Discovery of the InlA and InlB dependent mechanisms by which *L. monocytogenes *is thought to transgress the intestinal and placental barriers has resulted in a critical re-evaluation of mice and other traditional small animal models of listeriosis [24]. *L. monocytogenes *invasion proteins InlA and InlB are members of the internalin family, proteins with leucine-rich repeats (LRRs) [172]. Experimental inoculations in a variety of animal species, in primary cell lines, organ explants and immortalized cell lines using *L. monocytogenes *wild type, *inlA*, *inlB *and *inlA/inlB *deletion mutations and an *inlA *expressing *L. innocua *mutant have provided compelling evidence for the roles of these bacterial proteins in mediating internalization of *L. monocytogenes *into nonphagocytic cells [24]. The N-terminal regions of InlA and InlB contain signaling peptides and LRRs [172]. The C-terminal region of InlA contains a conserved LPXTG motif that confers covalent binding to peptidoglycans on the bacterial cell surface, while InlB contains a G-W motif, resulting in non-covalent binding to lipoteichoic acids in the bacterial cell wall [172,173]. Notably, due to the non-covalent nature of the binding InlB can be liberated from the bacterial cell wall, a process that is thought to play intricate roles during infection [174].

The importance of InlA for *L. monocytogenes *entry into non-phagocytic cells was demonstrated in 1991 when *L. monocytogenes *InlA was shown to confer *L. innocua *the ability to enter human Caco-2 cells (origin: human epithelial colorectal adenocarnicoma) and this finding has since been repeatedly confirmed [172,175,176]. Analogously, InlB has been shown to be required for *L. monocytogenes *internalization into various cell types such as immortalized Vero (origin: African green monkey kidney), HeLA (origin: human cervical adenocarcinoma), and CHO (origin: Chinese hamster ovary) cells [173,177]. An *in vivo* role of InlA and/or InlB, which are both encoded by the *inlAB *operon, for *L. monocytogenes *infection was suggested by Gaillard et al. in 1996, using a *L. monocytogenes *EGDΔ *inlA/inlB *deletion mutant [36]. After oral or intravenous challenge of SPF female Swiss mice with 10^9 ^or 10^5 ^cfu of the *L. monocytogenes *EGDΔ *inlA/inlB *deletion mutant, Gaillard et al. detected bacterial concentrations in the liver that were considerably lower than after challenge with EGD wt, and estimated LD_50 _values after intraveneous challenge equaled 5 × 10^6 ^and 3.5 × 10^7 ^for EGD wt and EGDΔ *inlA/inlB*, respectively [36]. Gaillard et al. also showed a reduced ability of *L. monocytogenes *EGDΔ *inlA/inlB *to invade TIB-73 cells, an immortalized hepatocyte line derived from BALB/c mice [36].

### 7.1 Interactions between E-cadherin and InlA

E-cadherin (Ecad, Ca^2+ ^dependent selective hemophilic adhesion molecule) is a transmembrane adhesion protein that mediates cell-cell junctions on epithelial cells and plays essential roles during embryonic development [172,174]. Ecad is expressed on most epithelial cells, but seems to be frequently replaced by P-cadherin on tumor cells, which may caution against the use of certain tumor-derived cell lines for the study of E-cadherin expression [178]. Ecad expression on polarized epithelial cells is typically located in adherence junctions and on the basolateral surface [172]. E-cadherin was identified as InlA receptor in 1996, again using human Caco-2 cells [179]. LCAM, a chicken ortholog of human Ecad, which shares approximately 65% sequence homology with Ecad [180], has been shown to promote InlA dependent entry into transfected immortalized fibroblast cell lines S180 (origin: Swiss mouse sarcoma) and L2071 (origin: C3H/An mouse connective tissue), providing convincing evidence that Ecad is sufficient to promote *L. monocytogenes *entry into these nonphagocytic cells [177,179]. The mechanism of InlA-mediated entry has since been elucidated: interactions between InlA and Ecad, located in lipid rafts, trigger intricate intracellular cascades that lead to actin rearrangement and ultimately result in caveolin-dependent endocytosis [11,172,174,181,182].

Murine Ecad (mEcad) has been cloned and studied extensively, primarily due to the crucial role of this molecule in cell sorting and cell-cell recognition during embryogenesis [180,183]. mEcad and human E-cadherin (hEcad) share approximately 85% sequence identity [180]. Surprisingly, however, *L. innocua *expressing InlA appeared unable to enter murine NMe cells (derived from NMuMG cells, an immortalized line of mouse mammary epithelial cells [180,184]), even though these cells have been shown to express high mEcad levels [180]. Similarly, transfection of various immortalized cell lines with mEcad (a cDNA construct originally derived from murine F9 embryonal carcinoma cells (i.e., an immortal cell line derived from 129/Sv inbred mice) for structure/function studies [180,185-187]) failed to promote entry of the *L. innocua *mutant expressing InlA, even though this *L. innocua *mutant readily entered the same cell lines transfected with human Ecad or chicken LCAM [180]. The inability of mEcad to promote InlA-dependent entry has been linked to a P16E mutation in the first extracellular domain of mEcad [180]. The sequence of rat Ecad at position 16 of this domain, also a glutamic acid, was determined using immortalized NBT2 (origin: rat bladder carcinoma) cells [180]. Guinea pigs, rabbits and gerbils, on the contrary, harbor a proline at position 16 [12,180,188]. This information was determined using GPC16 cells (origin: guinea pig colorectal adenocarcinoma), rabbit corneal epithelial cell cultures, and gerbil primary intestinal epithelial cell cultures [12,180,188].

The *inlA *gene of *L. monocytogenes *strain EGD has been successfully "murinized", leading to considerably increased susceptibility of wild type mice to infection with the murinized *L. monocytogenes *strains [189,190]. Two point mutations (i.e., S192N and Y369S) located in the *inlA *gene of murinized *L.monocytogenes *strain EGD-InlA^m^, have been shown to lead to increased susceptibility of C57BL/6 and BALB/c mice to oral infection, resulting in an LD_50 _of approximately 5 × 10^7 ^CFU in C57BL/6 mice, while only approximately 20% of C57BL/6 mice challenged with 5 × 10^10 ^cfu of wild type EGD died [189,190]. An N259Y mutation in *L. monocytogenes *EGD *inlA *has also been shown to promote efficient infection of BALB/c mice after intra-gastric inoculation [190].

iFABP-hEcad transgenic mice, generated in a (C57BL/6 J × SJL/J) background, express hEcad under control of the *iFABO *promoter [149]. In these mice, hEcad expression is limited to the intestine while mEcad is expressed on all epithelial cells that express mEcad in wild-type mice. iFABP-hEcad mice showed 85% mortality after oral challenge with 5 × 10^10 ^CFU of *L. monocytogenes *strain EGD while 100% of wild-type mice of the same murine strain and challenged under identical conditions survived [149]. This translates into LD_50 _values of > 10^10 ^and approx. 10^10 ^for wild type and iFABP-hEcad mice, respectively (Table 1). Notably, 100% of wild type and transgenic mice challenged with the same dose of *EGD*Δ*inlA*, an *inlA *knock-out strain of *L. monocytogenes *EGD, survived [149]. These results are comparable to those obtained for guinea pigs challenged with 5 × 10^11 ^CFU of EGD or *EGD*Δ*inlA*, respectively, with the LD_50 _for wt EGD in guinea pigs equaling approx. 10^11 ^cfu [149]. Like (starved) wild type mice, (starved) gnotobiotic Fabpi-hEcad mice, generated by back-crossing iFABP-hEcad transgenic mice to mice of strain C57BL/6 J and reared under gnotobiotic conditions, did not exhibit mortality after oral inoculation with 10^9 ^cfu of EGD or *EGD*Δ*inlA*, even though some clinical symptoms were reported [191]. Notably, in gnotobiotic Fabpi-hEcad mice, EGD was detected in villus enterocytes and the underlying lamina propria while such observations were not made in wild type mice [191]. However, *L. monocytogenes EGD*Δ*inlA *strains were able to infect the spleen of gnotobiotic Fabpi-hEcad mice, albeit at levels below those observed in Fabpi-hEcad mice infected with EGD, strongly indicating the presence of alternate infection pathways in these mice [191].

E16PmEcad knock-in mice^a ^carry a point mutation at position 16 of the first extracellular domain of murine E-cadherin that changes the glutamic acid at this position to a proline (i.e., E16P mutation) [12]. E16PmEcad mice are homozygous and therefore exclusively express "humanized" E-cadherin on all tissues where murine E-cadherin is expressed in wild-type mice [12]. In (starved) E16PmEcad mice experimental inoculation with 10^9 ^cfu EGD, InlA-dependent crossing of the intestinal barrier has been observed [12]. Unfortunately, information as to whether deaths were observed with this challenge dose was not provided and the available data do not permit calculation of LD_50 _values [12].

### 7.2 InlB and its three receptors

The hepatocyte growth factor receptor (HGFR or MET), complement component 1 Q subcomponent-binding protein (gC1qR) and glycosaminoglycans are known receptors for InlB and have been shown to mediate *L. monocytogenes *entry into a broad variety of host cells [192-194]. gC1pR is a ubiquitous, multiligand binding glycoprotein and acts as receptor for complement C1 [172]. Specific interactions between gC1pR and InlB have been documented and gC1pR and MET appear to act synergistically [173]. MET, a receptor tyrosine kinase, is expressed on a wide variety of epithelial and endothelial cells and binds hepatocyte growth factor (HGF) with high affinity [192-194]. MET is important for normal embryonic development, but also appears to play a key role in oncogenesis [173]. InlB binding leads to MET activation through transient phosphorylation of its multiple docking sites [173]. A complex intracellular cascade (see for instance [173,174] for a review) subsequently leads to actin reorganization, manifested as "membrane roughing", and culminates in clathrin-mediated endocytosis [173,174]. MET activation through HGF binding is considerably enhanced by the presence of glucosaminoglycanes (GAG) on the cell surface [173]. Notably, the C-terminus of InlB has also been shown to bind GAGs on the host cell surface, and InlB-dependent invasion is impeded in the absence of GAG [173,194].

The importance of InlB for *L. monocytogenes *infection has been demonstrated in-vivo and in-vitro. Bacterial counts in the liver and spleen of BALB/c mice, intravenously inoculated with 3 × 10^3 ^cfu of EGDΔinlB, were significantly (*p *< 0.02) lower 72 h after inoculation than in mice inoculated with the same dose of EGD wt, strongly suggesting a role of InlB-dependent colonization of these organs during *L. monocytogenes *infection in mice [45]. Surprisingly, however, such InlB dependence was neither observed in (starved) guinea pigs inoculated intragastrically with 10^10 ^cfu (plus calcium carbonate) or intravenously with 10^6 ^cfu of the same *L. monocytogenes *strains, nor in rabbits inoculated intravenously with 10^6^-10^7 ^cfu of the same strains [45]. Species-specific differences in internalization efficacy were confirmed in-vitro using immortalized cell lines of human, mouse, rabbit, and guinea pig origin^b ^[45]. Nearly all tested cell lines from guinea pigs and rabbits expressed both MET and gC1qR [45]. However, in guinea pig derived cell lines neither InlB nor human HGF induced membrane ruffling, a prerequisite for InlB dependent internalization [45]. In rabbit-derived cells, the presence of human HGF, but not InlB, induced such membrane ruffling [45]. Surprisingly, transfection of human MET conferred permissiveness in guinea pig and rabbit cell lines, indicating likely species-specific receptor differences [45]. In gerbils, both InlA and InlB mediated entry pathways appear to be functional as determined using primary intestinal cell cultures and confirmed *in vivo* using pregnant gerbils, but calculation of LD_50 _values has so far unfortunately not been possible [12]. Gerbils may conceivably represent an attractive rodent model of *L. monocytogenes *infection [12]. As perhaps expected for a rodent species, gerbils appear to cluster with mice and rats in dendograms based on either the Ecad or the MET sequence, but may form a separate phylogenetic clade in the Ecad phylogeny [12].

Other, currently unknown species-specific differences may exert so far unrecognized impacts on *L. monocytogenes *infections. Various *L. monocytogenes *virulence proteins such as listeriolysin O or ActA have been shown to play crucial roles in the *L. monocytogenes *infection pathway, while the role of other proteins is just beginning to be recognized [24]. *Vip*, for instance, encodes another *L. monocytogenes *LPXTG surface protein, which interacts with endoplasmatic reticulum resident chaperon Gp96 [195]. Guinea pigs as well as iFABP-hEcad transgenic mice have been shown to harbor lower bacterial loads in the liver, intestine, lymph nodes and spleen when orally inoculated with 10^10 ^cfu or 5 × 10^9 ^cfu, respectively, of *L. monocytogenes *EGDΔvip than when inoculated with the same doses of wt EGD, and similar observations have been made in BALB/c mice after intravenous inoculation [195]. Potential differences in vip-Gp96 interactions in other animal species, however, remain yet-to-be determined. As the interactions between *L. monocytogenes *and its host are increasingly being understood, new criteria for the adequacy of animal models of human listeriosis will likely continue to emerge.

## 8. Other animal models of non-pregnancy-associated listeriosis

The mouse model has been the most commonly used animal model of *L. monocytogenes *infection due to its size, ease of handling, and relatively low economic cost [11,23]. Moreover, the commercial availability of many reagents for immunological studies, the extensive available data on mouse physiology, anatomy, embryogenesis and immunology, and the existence of well-characterized inbred mouse strains with known physiological and immunological characteristics have contributed to the immense popularity of the non-pregnant mouse model of listeriosis. However, as discussed below, other small animal species have also occasionally been used to study certain aspects of *L. monocytogenes *infection and immunity in non-pregnant animals (Table 2). Since the species-specific differences in InlA-Ecad and InlB-MET interactions have been discovered, the value of mice, as well as rats, guinea pigs and rabbits as models for listeriosis in humans has been questioned [11]. Non-human primates or unconventional small-animal models, such as gerbils, may ultimately prove to be superior models of listeriosis, but only limited data on *L. monocytogenes *infections and immune responses in these animal species are so far available and ethical, economic and practical considerations, especially for non-human primates, will necessarily limit the number and size of studies that can be performed in these animal species.

**Table 2:** Summary of benefits and limitations of the different animal models (see text for details and references)

	Non-pregnant animals			Pregnant animals		
**Species**	**Popularity**	**Benefits**	**Limitations**	**Popularity**	**Benefits**	**Limitations**

mouse	high	- very well characterized - economical - large sample size possible - inbred strains available - immune reagents available - proven ability to model bacterial strain variability	- mutation in receptor for InlA (Ecad) affects entry into enterocytes - susceptibility to infection differs by mouse strain - body size limits some manipulations	moderate/high	- same as for non-pregnant animals - similarities to human - placentation well characterized	- mutation in receptor for InlA (Ecad)affects crossing of placental barrier - small body size

rat	moderate	- well characterized - economical - large sample size possible - inbred strains available - immune reagents available - body size optimal for certain manipulations - proven ability to model bacterial strain variability	- mutation in receptor for InlA (Ecad)affects entry into enterocytes - quite resistant to infection	moderate	- same as for non-pregnant animals - similarities to human placentation - body size optimal for certain manipulations	- mutation in receptor for InlA (Ecad) affects crossing of placental barrier - quite resistant to infection

rabbit	moderate/low	- well characterized - economical - large sample size possible - commonly used to generate antibodies - quite susceptible to infection	- InlB receptor (MET) polymorphism affects entry into cells such as hepatocytes	moderate/low	- same as for non-pregnant animals - similarities to human placentation - body size optimal for certain manipulations	- mutation in InlB receptor (MET) affects crossing of placental barrier

guinea pig	moderate	- well characterized - economical - large sample size possible - body size optimal for certain manipulations - ability to model bacterial strain variability	- InlB receptor (MET) polymorphism affects entry into cells such as hepatocytes - quite resistant to infection - pathological lesions often limited to myocardium	high	- same as for non-pregnant animals - similarities to human placentation - body size optimal for certain manipulations	- mutation in InlB receptor (MET) affects crossing of placental barrier - quite resistant to infection

gerbil	increasing	- quite susceptible to infection - functional receptors for InlA (Ecad) and InlB (MET)	- not very well characterized - no immune reagents - small body size less economical	increasing	- same as for non-pregnant animals	- same as for non-pregnant animals

chinchilla	low	- highly susceptible	- not well characterized - *inlA *&*inlB *receptor sequences unknown - no immune reagents less - economical	low	- same as for non-pregnant animals	- same as for non-pregnant animals

hamster	low	- economical	- resistant to infection - *inlA *&*inlB receptor *sequences unknown small body size	low	- same as for non-pregnant animals	- same as for non-pregnant animals

primate	moderate	- close phylogenetic relationship to humans	- ethical and economic considerations - limited sample size - no immune reagents	moderate	- same as for non-pregnant animals	- same as for non-pregnant animals

### 8.1. Non-pregnant rat models

Non-pregnant rats have been used repeatedly as models of *L. monocytogenes *infection, and rat antibodies have been used in mice to study the immunology of listeriosis [25,33,196-203]. Formal LD_50 _values after oral inoculation of adult rats are not available in the literature, but rats appear to be relatively resistant to infection. For example, juvenile rats orally inoculated with 10^2^-10^9 ^cfu of a *L. monocytogenes *4b strain developed dose-dependent invasive infection as measured by the presence of culturable bacteria in internal organs, but did not exhibit clear clinical symptoms or death [128,197]. Notably, treatment with cimetidine, a histamine receptor agonist that inhibits acid production in the stomach, significantly (*p *< 0.05) increased the probability of invasive infection in rats inoculated with less than 10^9 ^cfu, but did not significantly (*p *> 0.05) affect bacterial counts in the liver or spleen of infected animals, and bacterial counts in the organs did not appear to be dose dependent (*p *> 0.05) [197]. LD_50 _values for infant rats inoculated 3 days post-partum and juvenile rats, inoculated 13 days post-partum, determined after subcutaneous injection of an *L. monocytogenes *serotype 4b strain, equaled 6 × 10^5 ^and 2.5 × 10^7^, confirming the relatively high infectious dose for rats [204]. Rats have also for instance been used to study the impact of the gut microflora on susceptibility to infection [202]. In these experiments the presence of a normal gut microflora appeared to be somewhat protective of *L. monocytogenes *infection [202]. Gnotobiotic rats orally inoculated with 2 × 10^9 ^cfu of a *L. monocytogenes *serotype 1 strain exhibited weight loss, splenomegaly and histopathological lesions, and bacteria were readily recovered from the spleen and liver, even though rats appeared clinically healthy [202]. In conventionally reared rats or gnotobiotic rats switched to conventional feed during the experiment, however, *L. monocytogenes *appeared to be efficiently cleared from internal organs [202]. Rat ligated intestinal loop systems have been used to study early intestinal steps *of L. monocytogenes *infection - even though the Ecad receptor differences discussed above have since raised concern about the relevance of this system for modeling human infection [11,25,33]. Analogous to mice, rat models of *L. monocytogenes *infection have been used to study immune responses to infection [201]. Young rats have also been used to establish rat models of meningitis, and to evaluate the impact of different therapeutic regimens in these models [200]. For instance, in one of these experiments, rats were inoculated by intracisternal injection of 5 × 10^4 ^- 1 × 10^5 ^cfu of an *L. monocytogenes *4b strain and developed depression, weight loss and meningitis within one day of inoculation [200].

### 8.2 Non-pregnant guinea pig models

Non-pregnant guinea pigs have been used as models for listeriosis, even though inoculations have also often been performed in pregnant guinea pigs [16,53,205-208]. Guinea pigs appear to be somewhat more resistant to infection than most strains of mice, irrespective of the inoculation route [23]. High numbers of bacteria appear to be required to install infection in non-pregnant guinea pigs. LD_50 _values of about 10^11 ^cfu and greater than 10^8 ^cfu have been reported for oral and intraperitoneal inoculation, respectively [149,205], and for this reason, a standard dose of 10^10 ^cfu of *L. monocytogenes *EGD has been suggested for oral inoculation of guinea pigs, compared to doses of 10^8 ^- 5 × 10^9 ^cfu for mice [209]. Guinea pigs appear most susceptible to inoculation through the intracardial route, with an LD_50 _value of 1.2 × 10^5 ^cfu, while intravenous and intraperitoneal LD_50 _values appear to be high, in the range of 10^7 ^- 10^8 ^cfu and > 2.5 × 10^8^, respectively [205].

Other infection endpoints than death have occasionally been used in guinea pig studies and guinea pigs have for instance been used to identify differences in infectious doses among *L. monocytogenes *strains after oral inoculation [210]. Infectious doses of 10^7^-10^8 ^cfu after oral inoculation of juvenile male guinea pigs with an *L. monocytogenes *strain carrying a point mutation in *inlA*, and approximately 1 log lower infectious doses for inoculations with outbreak strains without the *inlA *mutation, have been reported [210]. Other researchers have also reported the ability to reproduce differences in *L. monocytogenes *pathogenicity among strains using orally infected non-pregnant guinea pigs [211]. However, infectivity again appeared to be relatively low. Rolgaard et al., for instance, gave two doses of 2 × 10^10 ^cfu each to the animals, one day apart [211]. He noted no deaths in response to these inoculations, and the occurrence of clinical symptoms such as depression and ruffed coat in only a few of the animals, on the day of euthanasia, even though bacteria could be harbored from the organs of most animals [211]. Oral inoculations of (starved) guinea pigs in the presence of calcium carbonate with 10^10 ^cfu of different *L. monocytogenes *strains led to reduced weight gain but did not cause noticeable clinical symptoms, perhaps again indicating a comparably low susceptibility to infection, even though the observation of clinical symptoms is dependent on the time of follow-up after inoculation [212]. Similar observations were also made in a study to evaluate the potential impact of food matrix on infectivity [213]. Pang and Matthews did not detect any clinical symptoms in starved, orally challenged guinea pigs, even if infectious doses of 10^8 ^cfu of a *L. monocytogenes *serotype 4b strain were used, and differences in bacterial loads in internal organs between food matrices were also not detected [213]. Geriatric guinea pig models have been used to evaluate the impact of immune modulation in elderly animals [21]. However, intragastric inoculation with 2.5 × 10^8 ^cfu of a *L. monocytogenes *serotype 4b strain again failed to produce death in the geriatric guinea pigs, and the presence of clinical symptoms was not described [21].

### 8.3 Non-pregnant rabbits as models of listeriosis

Rabbits appear relatively susceptible to infection with *L. monocytogenes*, even though LD_50 _values have not been formally calculated [23]. Rabbits have typically been used to generate anti-*Listeria monocytogenes *antibodies and to study immune responses to *L. monocytogenes *infection [214-216]. However, rabbits have occasionally also been used to model other aspects of *L. monocytogenes *infection, for instance to evaluate therapies for listeric meningitis [217]. Scheld et al., for example, inoculated animals with relatively high doses of 10^7 ^cfu of a *L. monocytogenes *strain from a human meningitis case by injection into the cisterna, and used changes in bacterial concentrations in the spinal fluid to evaluate the efficacy of different chemotherapies [217]. Abscess formation after subcutaneous inoculation of rabbits has also been reported [23].

### 8.4 Other non-pregnant rodent models

Nontraditional rodent species such as gerbils, chinchillas and lemmings, have occasionally been used as models of *L. monocytogenes *infection [23]. Chinchillas appear to be highly susceptible to oral inoculation, but seem to have been rarely used due to economic and practical considerations [23]. Chinchillas and gerbils have been used successfully to model listeric rhombencephalitis in animals with prolonged otitis media and bacteremia [218]. Gerbils were inoculated with 10^3 ^or 10^5 ^cfu of *L. monocytogenes *EGD by percutaneous injection into the superior chamber of the middle ear bulla [218]. Animals inoculated with high doses succumbed to disease 4-7 days after inoculation, while animals inoculated with low doses survived for 6-12 days [218]. Most gerbils exhibited behavioral changes, and typical neurological symptoms such as circling, ataxia, and paresia were observed at relatively late stages during infection [218]. Gerbils have also recently been used to model oral inoculation, even though only a relatively small number of animals were used and inoculation of 10^9 ^cfu of *L. monocytogenes *EGD did not appear to cause severe clinical symptoms in these animals [12]. Hamsters have rarely been used as models of listeriosis, but have been reported to be comparably resistant to infection [23]. Voles, on the contrary appear to be fairly susceptible to intra-peritoneal infection, but have rarely been used due to practical constraints on availability [23].

### 8.5 Non-human primates as models of listeriosis

Only a small number of experimental studies in non-human primates have been reported, primarily in pregnant animals [13,14]. However, Farber et al. inoculated non-pregnant cynomolgus monkeys with between 10^5 ^and 10^9 ^cfu of *L. monocytogenes *Scott A or a *L. monocytogenes *serotype 4b isolate from a food source, suspended in sterile milk, and only detected mild clinical symptoms such as fever, irritability, inappetence and in some cases diarrhea, primarily in animals challenged with high doses [15]. Interestingly, no marked differences between the two *L. monocytogenes *strains and no impact of treatment with antacids were observed [15]. One ape was challenged twice, approximately 8 weeks apart, with 10^9 ^cfu of *L. monocytogenes *Scott A, and fecal shedding in this animal after the second inoculation appeared shorter than in monkeys dosed once, potentially indicating some protective effect of prior exposure [15]. A small number of other experimental inoculations of non-human primates have also been reported, establishing for instance that apes appear to be relatively resistant to ocular or aerosol inoculation, even though transient ocular symptoms or febrile infection, respectively, could be invoked in these animals [23,115].

## 9. Animal models of pregnancy-associated listeriosis

Pregnancy significantly increases susceptibility to *L. monocytogenes *infection, regardless of the animal species or route of inoculation [23]. Similar to observations in non-pregnant animals, mouse strain specific differences in susceptibility of pregnant mice to *L. monocytogenes *infection have been reported, especially upon challenge with high doses [219]. Resistant mice strains exhibit a decreased risk of fetal resorption, lower mortality, and reduced bacterial loads in the liver and spleen as compared to susceptible mice strains [219]. The biological determinants of these differences, however, are currently still largely unclear.

The immunological, anatomical and physiological determinants of the increase in *L. monocytogenes *susceptibility during pregnancy have been subject to intense study, but have so far still only partially been resolved. Pregnancy is associated with immunological changes that result in a shift in the Th1/Th2 cytokine balance to favor Th-2 mediated, humoral responses [219]. Th-1 mediated cellular immune responses, primarily mediated through interferon gamma (INF*γ*) and tumor necrosis factor alpha (TNF*α*), are crucial for successful control of infections with intracellular pathogens such as *L. monocytogenes*, but appear significantly down-regulated during pregnancy [219]. Mouse models have been used extensively to study immunological changes during pregnancy, encompassing both physiological changes needed to protect the fetus and responses elicited following infection. As shown in comparative studies of pregnant and non-pregnant female mice of the BALB/c strain, T-cell mediated immune responses are significantly impaired during pregnancy and systemic INF*γ *levels are downregulated while IL-10 levels are upregulated [219]. Following *L. monocytogenes *infection, increased systemic levels of TNF*α *and IL-6 were detected in pregnant BALB/c mice compared to non-pregnant female BALB/c mice, while IL-8 ortholog Chemokine (C-X-C motif) ligand 1 (CXCL1, previously called KC) appeared downregulated, indicating a likely role in the diminished ability to control *L. monocytogenes *infection [219]. Mouse models have also been instrumental in elucidating pregnancy-associated immunological changes in specific tissues. In the liver of pregnant mice of the BALB/c strain, for instance, transcription of TNF*α*, INF*γ *and inducible Nitric oxide synthase (iNOS) is decreased after experimental inoculation with *L. monocytogenes *as compared to non-pregnant female mice [219]. The ability of the immune system to control *L. monocytogenes *replication in the liver, spleen and other organs therefore appears to be severely hampered during pregnancy.

The intricate immunological and physical roles played by the placenta in shielding the fetus from infection are slowly being revealed [219]. The murine placenta appears to represent a transient component of the innate immune system [220]. Comparative studies in knock-out mice identified a crucial role of colony stimulating factor 1 (CSF-1) in the control of *L. monocytogenes *infection [220]. After experimental inoculation with 1 × 10^4 ^cfu of *L. monocytogenes *EGD, CSF-1 induced expression of KC and macrophage inflammatory protein 2 (MIP-2) in the trophoblast resulted in recruitment of neutrophils, the predominant immune cells in the pregnant placenta [220]. Notably, wild type mice were able to control the infection by day 3 post inoculation, and carried their litters to term, while all knock-out mice aborted [220]. The placenta likely also encompasses other components of the innate immune system. For example, Toll-like receptors (TRLs) 2 and 4, components of the innate immune system that recognize the surface protein LPS present on the surface of gram-positive bacteria, are expressed on human placentas and a role in host defense against *L. monocytogenes *during pregnancy appears likely [221,222].

The placenta may represent an important *L. monocytogenes *harborage site during pregnancy, and may play a direct role in mediating increased susceptibility to infection. For example, inoculation of female non-pregnant and pregnant mice of strain CD1 through the intragastric route with 3 × 10^7 ^cfu *L. monocytogenes *10403S (serotype 1/2a) or a serotype 4 non b strain resulted in (extrapolated) LD_50 _values of approx. 1.5 × 10^8 ^cfu in pregnant animals [223]. None of the non-pregnant animals inoculated with 10^8 ^cfu died [223]. Surprisingly, bacterial loads in the colon, spleen and liver after oral inoculation differed between the *L. monocytogenes *strains, but in general no significant (*p *> 0.05) difference in bacterial loads among pregnant and non-pregnant animals were observed [223]. However, embryo, yolk sack and decidual tissue frequently harbored high number of bacteria, and infection of decidual tissues appeared to be correlated with increased maternal mortality [223].

Many details about the mechanisms by which *L. monocytogenes *crosses the placental barrier and causes abortion or stillbirth remain yet to be fully understood. Abortion is a common outcome of listeriosis in humans, monogastric mammals as well as ruminants, regardless of the type of placentation [23,55]. Ruminants, pigs and horses have an epitheliochorial placenta. The placenta of carnivores is an endotheliochorial placenta. Primates, rodents and lagomorpha, on the contrary, share a hemochorial placenta, characterized by direct contact between fetal chorion and maternal capillaries. Importantly, fewer cellular layers between fetal chorion and maternal capillaries generally result in more efficient transfer of nutrients and other macromolecules including immunoglobulins. The placentas of humans and mice are both of discoidal type and have been studied and compared extensively, primarily to study physiological and pathological processes during human pregnancy. Especially during the third trimester of gestation, human and murine placentas share considerable similarities in structure, function and likely also in the embryonic origin of placental cells [224]. However, some anatomical differences between the two species exist. For instance, in the murine placenta the spaces that contain the maternal capillaries appear considerably more maze-like than in human placentas while the fetal capillaries have a porous instead of continuous endothelium that facilitates exchange of small molecules (see [224] for a detailed comparison). Proteomic and transcriptomic comparison of microdissected human and murine placentas, focusing on the regions of materno-fetal exchange (i.e., villous tree in humans and labyrinth in mice), revealed 7000 orthologous genes of which 70% were co-expressed in both mice and humans [225].

In the placentas of women with listeriosis, as well as in the placentas of naturally or experimentally inoculated animals, *L. monocytogenes *can be detected in the intervillous spaces, the villous core, and the trophoblast [23,24]. These observations, paired with the absence of bacteria from the connective tissue of the chorion in human placentas, led to the hypothesis that *L. monocytogenes *infection occurs through the transplacental route [24]. *L. monocytogenes *is thought to cross the placental barrier at the interface of syncytiotrophoblast and fetal blood vessel [12]. On the contrary to observations in mice and guinea pigs, crossing of the human placenta appears to be both InlA and InlB dependent [12]. *In vivo* experiments in pregnant gerbils demonstrated that intravenous infection with 2 × 10^6 ^cfu *L. monocytogenes *EGD consistently led to fetal infection, while EGD *inlA*, *inlB *and *inlA/B *deletion mutants all showed significantly (*p *< 0.05) reduced placental invasion and fetal infection based on competitive indexing [12]. However, competitive indexing results appeared to vary somewhat among the deletion mutants and all deletion mutants appeared able to invade placenta and fetus, likely signifying the presence of alternate infection routes [12]. Surprisingly, in wild type mice and guinea pigs, invasion of the placenta appears to be neither InlA nor InlB dependent, but the placenta of E16PmEcad knock-in mice that express humanized Ecad in all organs, appears more than one order of magnitude more permissive to *L. monocytogenes *EGD than to the deletion mutants [12]. The exact mechanisms by which InlA and InlB dependent entry pathways interact in the placenta are currently unclear. Notably, cell-to-cell spread has also been shown to play a key role in fetal infection, at least in guinea pigs [53]. A variety of different infection pathways therefore appear capable of potentially leading to fetal infection, and species-specific differences likely impact fetal infections in different species.

Studies in pregnant animals differ considerably in the timing of inoculation relative to gestation, the length of follow-up after inoculation, and the study endpoint. Non-human primates and guinea pigs have received increasing attention in recent years, even though the adequacy of the guinea pig model has been critically re-evaluated since the species-specificity of the InlB-MET interactions have been elucidated [11,12]. Gerbils may represent promising rodent models of pregnancy-associated listeriosis, but the data available for this species are currently limited.

### 9.1 Non-human primates as models of pregnancy- associated listeriosis

A non-human primate model of *L. monocytogenes *induced stillbirth has been developed [14]. This animal model may potentially be the most appropriate model for human disease due to the close phylogenetic relatedness between humans and apes, but ethical, economic and practical considerations have thus far limited the number of studies and replicates available as well as the sample size. Stillbirth, premature birth as well as normal birth were observed in rhesus monkeys inoculated orally during the last trimester of gestation with 10^2^-10^10 ^cfu of *L. monocytogenes *strain Scott A, with another serotype 1/2a strain, or with 4b strains [13,14]. The inoculum was delivered in whipping cream (i.e., 30% milk fat), "half-and-half" (i.e., 11% milk fat) or skim milk (i.e., < 1% milk fat) - with the latter two food vehicles abandoned after initial testing because infectivity appeared to be the highest in whipping cream [13,14]. The respective dose required to induce stillbirth in 50% of non-human primates has been estimated to fall in the range of 10^6^-10^8 ^cfu *L. monocytogenes*, averaged across strains, gestation days and using whipping cream as food vehicle [13]. Clinical symptoms were not reported in any of the inoculated pregnant monkeys and *L. monocytogenes *was not isolated from all aborted fetuses [13,14].

### 9.2 Guinea pigs as models of pregnancy- associated listeriosis

Pregnant guinea pigs have repeatedly been used to model listeriosis, using a variety of different endpoints of infection [53,206,208]. Similar to observations in non-pregnant guinea pigs, pregnant guinea pigs appear to be relatively resistant to *L. monocytogenes*. For instance, pregnant guinea pigs inoculated with up to 10^8 ^cfu of a *L. monocytogenes *serotype 1/2a strain through the oral route were not reported to exhibit clinical symptoms, even though 20 of the 22 dams inoculated with 10^6 ^or more cfu harbored *L. monocytogenes *in the liver and spleen [16]. Noticeably, the occurrence of stillbirths appeared to be dose-dependent [226]. On average, 11% (1/9), 30% (3/10) and 75% (3/4) of the 23 dams inoculated with doses of 10^6^, 10^7 ^and 10^8 ^cfu aborted, and the average time interval between inoculation and abortion decreased from 20 to 10 days as the inoculation dose increased [227]. The occurrence of apoptosis in the placentas of these guinea pigs also appears to be dose-dependent [208]. The dose required to cause stillbirth in 50% of guinea pigs has been approximated at 10^7 ^cfu using an *L. monocytogenes *serotype 1/2a strain delivered orally in commercial heavy whipping cream [16]. Noticeably, mortality of fetuses in a given litter ranged from 0 to 95% and appeared to be dose-dependent [16]. Surprisingly, however, the level of bacterial invasion in the maternal liver did not appear to be a reliable predictor of fetal mortality [16].

Relatively high infectious doses seem to be needed to cause fetoplacental infection in guinea pigs [206]. Bakardjiev et al., for instance, reported that 10^6 ^cfu of *L. monocytogenes *strain 1043S had to be given intravenously to cause maternal and fetoplacental infection [206]. When guinea pigs were inoculated with 2 × 10^7 ^cfu, *L. monocytogenes *appeared to accumulate in the placenta and placentas contained multifocal inflammatory lesions in the labyrinth as well as surrounding the maternal blood vessels [206]. Most, but not all fetuses of a given dam became infected during challenge with high doses [206]. In another study, pregnant guinea pigs (gestation day 42-52, representing 2^nd ^to 3^rd ^trimester of gestation in humans) were inoculated intravenously with 10^9 ^cfu *L. monocytogenes *1043S and *L. monocytogenes *was detected in the livers of all fetuses 24 h post inoculation [53]. Clinical symptoms in the pregnant guinea pigs, however, were not reported [53]. Notably, *L. monocytogenes *strain - specific differences in the ability to invade maternal, placental and fetal tissues of experimentally inoculated guinea pigs after oral inoculation in whipping cream have been described, indicating the likely importance of *L. monocytogenes *strain-specific properties for infection in pregnant as well as non-pregnant animals [228].

### 9.3 Other rodent models of pregnancy- associated listeriosis

As discussed above and similar to the use of geriatric mouse models to understand immunological responses [20], mice have repeatedly been used to understand immune responses to *L. monocytogenes *infection during pregnancy (see for instance [229,230]). Pregnant BALB/c mice, challenged through the intraperitoneal route with 10^5 ^cfu *L. monocytogenes *EGD, have also been used to demonstrate a protective role of CpG oligodeoxynucleotides (CpG ODN), short single-stranded synthetic DNA molecules that contain CpG motives which stimulate innate immune responses through TLR-9, on *L. monocytogenes *infection [231]. Pregnant mice appear to be considerably more susceptible to infection after oral inoculation than non-pregnant mice, but susceptibility in pregnant mice also differs with *L. monocytogenes *strain [223]. Comparison of different mice strains, gestation days at inoculation, and days of sacrifice identified BABL/c mice as the most adequate strain and inoculation on gestation day 14 as the optimal timing [219]. Non-pregnant BALB/c mice appeared to clear bacteria more rapidly after intravenous inoculation with 2.5 × 10^4 ^cfu of *L. monocytogenes *EGD than pregnant mice inoculated on varying days of gestation, with mean durations of infection equaling 7 and 14 days, respectively [232]. Clinical symptoms in the inoculated dams were not reported [232].

Increased susceptibility during pregnancy has also been demonstrated in rats after subcutaneous infection with a *L. monocytogenes *serotype 4b strain, with LD_50 _values equaling 10^8 ^and 10^9 ^cfu for pregnant and non-pregnant rats, respectively [204]. Susceptibility appeared to be the greatest on day 16 of gestation, while infection on gestation day 9 appeared to result in resorption of fetuses but survival of the dams [204]. Gerbils have recently been used as experimental models of infection and the data appears somewhat promising, but so far the data available on *L. monocytogenes *infection in this species is very scarce [12]. Ultimately, it appears that the final verdict regarding the most adequate model of *L. monocytogenes *infection during pregnancy has to be postponed until the pathophysiology and immunology of *L. monocytogenes *infection during pregnancy has been elucidated in sufficient detail to permit comprehensive evaluation of the strengths and limitations of different animal models. It seems for instance that the intricate and interdependent roles of InlA and InlB in the transgression of the placental barrier have to be understood completely and that the nature of alternate infection routes that appear to permit infection *in vivo* has to be elucidated before a final, evidence-based decision about the choice of animal models can be made.

## 10. Geriatric models of listeriosis

Elderly and immunosuppressed individuals are at a particularly high risk of acquiring listeriosis [55]. Animal models specific to these population subgroups have been developed and geriatric models have occasionally been used to study infections with *L. monocytogenes*, as well as other pathogens such as *Salmonella, Staphylococcus aureus *or *Toxoplasma gondii *[233-239] but such models are very cost intensive and numerous questions about their relevance for human disease remain [240]. In geriatric patients as well as in aged laboratory animals, aging and underlying diseases are often intricately linked [241]. Old laboratory mice, for instance, are very commonly affected by underlying conditions such as hepathopathies, glomerulonephropathies, or neoplasies, and age-dependent changes in cells, tissues and organs often begin to develop before mice reach their median life expectancy [240]. Animal species differ in median life expectancy, in common geriatric diseases and conditions, and for mice the aging process appears to differ considerably among strains [240,241]. The determination of age equivalencies between humans and laboratory rodents is complicated by dynamically changing age relationships over the course of life, with one study quoting age equivalencies for 12, 45 and 70 year old humans as 1, 13 and 24 month old mice [241]. Clear definitions of geriatric models that are based on judicious selection of animal species and strains, age group and individual animals are therefore an indispensable prerequisite to permit meaningful inference for humans, but for many animal species, only limited knowledge of geriatric processes is currently available.

The immunological and physiological changes that determine the increased susceptibility of geriatric individuals to infection are only partially understood, but seem to be predominantly associated with functional defects in the lymphocyte-macrophage system [242]. T-cell mediated immunity is decreased in aged individuals, probably primarily due to decreasing numbers of naïve T cells, higher expression of prostaglandin E_2 _by macrophages, and intrinsic changes in naïve and memory T cells such as decreased IL-2 secretion and T-cell receptor expression, increased expression of suppressors of cytokine signaling 3, and defects in the T cell signaling pathway [243]. Phagocytic cells, including Kupfer cells, of geriatric individuals also generally appear to be impaired in their endocytic capacity [244]. Aged mice are more sensitive to LPS than younger animals, manifested as decreased LD_50 _values and increased expression of cytokines IL-1α, IL-6, IL-10 and TNF-α after LPS exposure [245]. Geriatric individuals therefore seem to differ from middle-aged adults in numerous ways. Importantly, nutritional factors such as vitamin E have been shown to enhance T cell-mediated functions in geriatric animals and humans, emphasizing the complexity of modeling geriatric disease [243].

Several studies have reported an increased susceptibility of geriatric animals to infection with *L. monocytogenes *[246]. Patel, for instance, inoculated 8 to 12 week and 24 to 28 month old backcrossed (A/Tru × C57Bl/6) mice with 10^3 ^to 10^4 ^cfu of *L. monocytogenes *strain EGD via the intravenous route, and detected higher bacterial loads in the liver and spleen of old mice [247]. In another study, Patel [144] found 24 month old (A/Tru × C57Bl/6) backcrossed mice inoculated with *L. monocytogenes *strain EGD more susceptible to infection than 8 month old mice of the same strain, with LD_50 _values equaling 1.6 × 10^5 ^and 4 × 10^6^, respectively. In transfusion experiments Patel determined that T-cells derived from geriatric (A/Tru × C57Bl/6) backcrossed mice were 100 fold less efficient at protecting naïve mice from infection with *L. monocytogenes *strain EGD than T-cells derived from younger animals regardless of the age of the recipient mouse, and geriatric mice appeared to produce approximately 10 fold fewer protective T-cells than younger animals [236]. Aged mice of the BALB/c strain were found to be increasingly susceptible to infection with *L. monocytogenes *after intravenous infection [233]. Notably, significant (*p *< 0.05) age-dependent differences after challenge with 10^5 ^cfu of *L. monocytogenes *were detected in mice as young as 11 month of age when compared to younger mice, but differences were considerably more pronounced in 18 than 11 month old mice [233]. Geriatric rats, 20 months of age, were more susceptible to pulmonary disease after intratreacheal inoculation with *L. mononocytogenes *strain 10403S than younger rats 2.5 months of age [234]. Notably, all geriatric rats died within 6 days of intratracheal challenge with 5 × 10^5 ^cfu of *L. mononocytogenes *strain 10403S, while 28% of younger rats were still alive 7 days after challenge, even though they succumbed to infection by day 8 post challenge [234].

Wu et al. [248] inoculated starved guinea pigs retired from breeding colonies (weighing approx. 1000 g) and starved younger guinea pigs (weighing 250-300 g) with 100 CFU of a *L. monocytogenes *serotype 4b strain via oral gavage. Overall, 15% (i.e., 6/39) of geriatric animals and 8% (3/37) of younger animals developed infection based on pathogen detection in the liver and spleen, but none of the animals succumbed to infection, and only occasional mild gastro-intestinal symptoms developed in any of the animals [248]. Treatment with vitamin E appeared to have a protective effect for both geriatric and younger animals [248]. Similar results have also been described by Pang et al. [21], who analyzed the impact of vitamin E on *L. monocytogenes *infection in approximately 2-year-old guinea pigs that had been retired from breeding colonies. Bruce et al. [235] reported somewhat impaired clearance of *L. monocytogenes *from the liver and spleen of vitamin D receptor knock-out mice compared to wild type mice of strain C57BL/6, again emphasizing the potential impact of nutritional factors.

Notably, on the contrary to the results presented above, some studies have reported an increased resistance of older mice to *L. monocytogenes *infection. Lovik et al. [237], for instance, reported an increased mean time to death (i.e., 6.7 vs. 4.7 days) and a decreased LD_50 _value (6.4 × 10^5 ^to 1.8 × 10^6 ^vs. 1.2 × 10^5 ^to 8 × 10^5^) for 22 to 30 months compared to 11 to 16 week old (A/Tru × C57Bl/6) backcrossed mice inoculated intravenously with *L. monocytogenes *strain EGD. Gervais et al. [249] studied the susceptibility of 18 to 22 month old (A/J × C57BL/6 J) backcrossed mice to *L. monocytogenes *infection and reported diminished subcutaneous inflammatory responses compared to younger animals. Surprisingly, macrophages and Kupffer cells isolated from the older mice exhibited increased in-vitro antilisterial characteristics compared to cells isolated from younger mice, again indicating increased resistance of older mice to *L. monocytogenes *infection [249]. Several fundamental questions about the relevance of current geriatric models for the study of listeriosis therefore clearly remain.

## 11. Conclusions and lessons learned

In conclusion, the pathophysiology and immunology of *L. monocytogenes *infection is increasingly being elucidated, but several key components remain to be explained that may ultimately revolutionize our thinking about the adequacy of animal models for modeling disease in humans. Species-specific differences in Ecad and MET have been identified, but their definitive roles in the pathophysiology of listeriosis remain yet to be determined. As discussed above, rabbits and guinea pigs both appear to harbor MET receptors that cannot be activated by InlB, but rabbits seem considerably more susceptible to infection than guinea pigs and histopathological lesions in non-pregnant guinea pigs often appear localized in the myocardium. Murine and rat Ecad both carry the Pro16Glu mutation, while guinea pigs, rabbits and chickens do not. Yet, rats and guinea pigs appear considerably more resistant to *L. monocytogenes *infection than most mouse strains. Many questions regarding susceptibility differences among mouse strains, age groups, and *L. monocytogenes *strains also so far remain unresolved.

Mouse models have been widely used for many decades and much of the currently available data on pathophysiology and immunology of *L. monocytogenes *infections has been generated in mice. However, recent findings cast doubt on the adequacy of this animal model, at least for certain aspects of infection. Novel, perhaps more appropriate animal models are currently being evaluated, but considerably more data has to be collected before the adequacy of these models can be evaluated comprehensively. Ideal animal models are biologically relevant, reliable, cost-effective, and sustain strict scientific and ethical scrutiny. Currently, no single animal model appears to fully meet all of these criteria and it appears that the most adequate animal model of human disease may have to be selected based on the specific aspect of infection to be studied, at least until more adequate animal models have been validated. A variety of animal species are susceptible to naturally occurring listeriosis, but the degree of susceptibility and the clinical manifestations appear to differ by species, physiological state, age group and *L. monocytogenes *strain. Perhaps with the exception of rhombencephalitis, invasive listeriosis appears to be an infection of immuno-compromised individuals - the young, the old, the pregnant and those with immune disorders appear to be at risk of developing disease. To date, it has been impossible to reliably reproduce the full spectrum of clinical disease in adult animals through experimental inoculation using relevant inoculation routes, regardless of the animal species, even when geriatric animals were used, multiple doses were given, or immune suppression was experimentally evoked in the animals. It thus appears that a more comprehensive understanding of the immunological determinants of susceptibility, as well as of the pathophysiology of infection, is needed before fully satisfactory animal models can be devised.

### 11.1 Consequences for modeling *L. monocytogenes *dose-response

Animal models have repeatedly been used to infer infectious or lethal doses for humans, estimates to be used in risk assessments or policy decisions. However, given the extraordinary variability in dose-response among animal species, the impact of *L. monocytogenes *strain-specific differences, and the numerous remaining questions about pathophysiology and immunology, it appears unclear which if any animal model is the most adequate to model human dose-response. Non-human primates may be the most relevant animal model for human dose-response, but so far only a limited number of experiments, restricted, due to ethical and practical considerations, to a small number of pregnant animals, *L. monocytogenes *strains, food matrices and only using application of a single dose (as opposed to multiple dosing), have been conducted. The available data with this animal model will only capture a limited amount of variability in dose-response relationships. Moreover, non-human primate dose-response models are currently not available for elderly, immune-compromised or neonatal (i.e., late-onset listeriosis) cases, which together constitute a considerable fraction of human cases. In the absence of other data, data collected in mice and other animal species may possibly be substituted to infer measures of variability among L. monocytogenes strains, species, genetic backgrounds and immune conditions and to understand basic properties of the immune responses elicited in response to infection. However, such data has to be diligently tied to epidemiologic data collected among humans, results have to be interpreted in the light of emerging data on species-specific differences, and the limitations of such inferences have to be clearly acknowledged. Comparative studies, combining evidence collected in more than one susceptible animal species such as non-human primates or gerbils, may allow for more meaningful inference about human dose- response. However, considerable differences in experimental design, disease outcome of interest, and genetic, physiological and immunological make-up of (often inbred) laboratory animals complicate comparisons across studies. Weight-of-evidence approaches may prove suitable to elucidate the impact of certain factors on dose response, such as for instance strain variability among mice. However, in the absence of a clear understanding of pathophysiology and immunology, weight-of evidence approaches may prove confusing or even misleading. The potential impact of various experimental conditions, such as the food matrix used for inoculation, the potential effect of antacids or starvation, the effect of repeated dosing - with short or long time intervals between doses -, the potential effect of microscopic lesions inflicted for instance during intragastric inoculation, and the true impact of *L. monocytogenes *strain - specific characteristics in different species or physiological stages, remain yet to be determined. However, such data will be vital to adequately evaluate the biological relevance of a given animal model for human disease.

In summary, much has been learned about *L. monocytogenes *since its first isolation from naturally infected rodents, but truly suitable animal models of infection are so far still missing, and many uncertainties remain when animal models are employed to model human infection.

## 12. Competing interests

The authors declare that they have no competing interests.

## 13. Authors' contributions

SD, KH and RP conceived and planned the study and participated in the decision to publish. KH reviewed the literature and KH and RP prepared the manuscript. All authors read and approved the manuscript.

## 15. Endnotes

^a^E16PmEcad mice were generated by crossing germline chimeras (i.e., chimeras between: i) transgenic CK35 embryonic stem (ES) cells - an ES cell line derived from murine strain 129/Sv [250]- that were genetically modified to express murine E-cadherin with the E16P mutation (i.e., 'humanized' E-cadherin), and ii) C57BL/6 blastocystes) with mice transgenic for the Cre site-specific recombinase that were generated in the BALB/c × C57B1/B6 background [12,251].

^b^i.e., HeLa (origin: human cervical adenocarcinoma), Nme (derived from NMuMG cells, an immortalized line of mouse mammary epithelial cells [180,184]), RK13 (origin: rabbit kidney cells), GPC16 (origin: guinea pig colorectal adenocarcinoma), 104 (origin: guinea pig fetal fibroblast) and JH4 (origin: guinea pig lung fibroblast) cells.
